# INTERMITTENT KETOGENIC DIETS EXTEND LIFESPAN AND ATTENUATE INFLAMMAGING IN MALE MICE

**DOI:** 10.1093/geroni/igac059.2671

**Published:** 2022-12-20

**Authors:** Zeyu Zhou, Kyoungmi Kim, Jon Ramsey, Jennifer Rutkowsky

**Affiliations:** University of California, Davis , Davis, California, United States; University of California, Davis , Davis, California, United States; University of California, Davis , Davis, California, United States; University of California, Davis , Davis, California, United States

## Abstract

A ketogenic diet (KD) has been shown to increase functional lifespan in male mice when fed isocalorically to a control diet (CD). However, it is not clear if intermittent ketogenic diets (IKD) that induce brief and recurring periods of ketosis would be as effective at prolonging lifespan. In the present study, 12-month-old C57BL/6JN male mice were fed an intermittent single day KD (KD fed on Mon, Wed and Fri each week, IK1) or 2-day KD (KD fed on 2 consecutive days each week, IK2). These two IKD regimes were selected to mimic the widely used alternate-day or 5:2 intermittent fasting approaches, without the caloric deficit. Our results showed that lifespan was significantly increased in both IK1 and IK2 groups, with IK1 showing a 13.6% and IK2 showing a 7.9% increase in median lifespan compared to the CD (p < 0.01). At 27 months of age, levels of circulating proinflammatory cytokines were significantly decreased in IK1 and IK2 mice compared to control mice. Also, levels of proinflammatory cytokines were significantly elevated in 27-month-old control mice compared to 12-month-old mice, while levels in IK1 and IK2 mice were not significantly altered with aging, and these effects were still persistent during days the animals were fed the CD. Unlike the continuous KD, health span measures were not altered in IK1 or IK2 mice at 26 months of age. In summary, IKDs that produce shorts episodes of ketosis can be effective at extending longevity in mice, possibly by attenuating systemic inflammation with aging.

